# From molecular networks to translational intervention: current progress in the mechanisms of gemcitabine resistance in bladder cancer

**DOI:** 10.3389/fimmu.2026.1860957

**Published:** 2026-05-29

**Authors:** Ming Jin, Zhenzhen Cai, Ming Bu, Jicheng Liu, Xiaojie Zhang

**Affiliations:** 1 Heilongjiang University of Chinese Medicine, Harbin, China; 2 Qiqihar Medical University, Qiqihar, China

**Keywords:** biomarker, bladder cancer, DNA damage repair, gemcitabine, resistance

## Abstract

Bladder cancer remains one of the most common malignancies of the urinary tract. Although the treatment landscape has expanded rapidly in recent years, gemcitabine still occupies a central position in intravesical treatment for non-muscle-invasive bladder cancer, in perioperative systemic therapy for muscle-invasive disease, and in platinum-based first-line regimens for advanced urothelial carcinoma. Yet the long-term benefit of gemcitabine is frequently curtailed by primary non-response or acquired resistance. In practice, this problem is often recognized only after radiographic progression or clear clinical deterioration has occurred. This review summarizes recent progress in bladder cancer therapy and translational research, with a particular emphasis on the biological basis and hierarchical evolution of gemcitabine resistance. We establish a 3-stage operational model of resistance, distinguishing: (1) early pharmacologic resistance driven by impaired drug uptake/activation or enhanced inactivation; (2) intermediate resistance driven by enhanced DNA damage repair, replication stress tolerance, and pro-survival autophagy signaling; and (3) late adaptive resistance driven by epithelial-mesenchymal transition (EMT), stemness maintenance, metabolic reprogramming, non-coding RNA-mediated epigenetic regulation, inflammatory microenvironmental remodeling, and extracellular vesicle-based intercellular transmission. These layers function as an interactive network, with sequential emergence under treatment pressure and parallel activation in context-dependent clinical settings. We stratify key mechanistic nodes (including the HYAL4-V1/CD44/JAK2-STAT3/CDA axis, AKR1C3, AP1M2-RAD54B, PRPF19-DDB1, AKT/mTOR signaling, Beclin-1-dependent autophagy, the MINCR/ZEB1/PHGDH axis, and IL-6-associated inflammatory states) by their clinical evidence quality and translational readiness, explicitly distinguishing preclinical discovery from clinically validated findings. Critically, most mechanistic findings remain at the preclinical or retrospective validation stage, with no markers yet approved for routine clinical use. Future work must prioritize longitudinal paired clinical samples, standardized analytic assays for dynamic biomarkers, and the integration of functional models (organoids, microfluidic systems), multi-omics technologies (single-cell sequencing, spatial transcriptomics), and liquid-biopsy approaches to translate mechanistic discoveries into clinically actionable predictive tools and therapeutic strategies.

## 1 Introduction

Bladder cancer is one of the most common urologic malignancies worldwide and a major source of long-term disease burden for patients and health systems alike. It ranks among the most frequently diagnosed cancers globally, and both incidence and mortality remain substantially higher in men than in women (1–4). Clinically, bladder cancer is a heterogeneous disease rather than a single entity. Non-muscle-invasive bladder cancer (NMIBC) constitutes most newly diagnosed cases. It is less lethal at presentation, but it is characterized by frequent recurrence, repeated endoscopic procedures, and prolonged surveillance. Muscle-invasive bladder cancer (MIBC), by contrast, is less common at initial diagnosis yet far more aggressive, with a much higher risk of metastasis and cancer-related death (2–5). These differences matter because they shape not only prognosis but also patterns of treatment exposure, cumulative toxicity, and the opportunities for resistance to emerge.

Over the past decade, the management of bladder cancer has entered a period of genuine transition. Molecular classification has refined our understanding of disease biology, immune checkpoint inhibition has altered the treatment algorithm for advanced disease, and bladder-preserving multimodality approaches have become more sophisticated (5–10). Even so, gemcitabine has not been displaced. It remains embedded in routine practice across multiple clinical scenarios, including intravesical therapy, neoadjuvant or perioperative systemic treatment, and cisplatin-based first-line therapy for advanced urothelial carcinoma (5, 7–10). This persistent clinical relevance is exactly why gemcitabine resistance deserves close attention. Once tumor sensitivity falls, the value of several standard regimens drops sharply, and treatment decisions become much more dependent on patient fitness, access to newer agents, and local practice patterns. In other words, understanding gemcitabine resistance is not a narrow pharmacology question. It is central to treatment sequencing, biomarker development, and precision management in bladder cancer.

In this review, we establish a 3-stage hierarchical model of gemcitabine resistance, stratify mechanisms by their clinical evidence strength and translational readiness, critically evaluate the barriers to clinical translation, and provide an actionable roadmap for advancing mechanistic discoveries into patient care. This framework addresses a critical unmet need in the field: to transform the growing body of preclinical resistance data into clinically useful guidance for both researchers and clinicians.

## 2 Advances in bladder cancer treatment and the clinical role of gemcitabine

The current bladder cancer treatment landscape has expanded dramatically in recent years. In NMIBC, intravesical therapy remains the cornerstone of disease management, with gemcitabine as a first-line agent for BCG-naive and BCG-unresponsive patients (7, 9). For MIBC, cisplatin-based perioperative chemotherapy (predominantly gemcitabine + cisplatin, GC) remains the standard of care for curative-intent treatment, alongside radical cystectomy and trimodality bladder preservation (5, 7, 8). In advanced urothelial carcinoma, the standard of care has evolved to include maintenance immunotherapy, biomarker-selected targeted therapy, and antibody-drug conjugates (ADCs) in earlier lines of treatment (7, 11–17). Even so, for cisplatin-eligible patients, GC remains the definitive first-line therapeutic backbone, with gemcitabine also widely used in cisplatin-ineligible patients and in combination with ADCs or immunotherapy (7, 12, 13). This enduring, cross-stage clinical role means that gemcitabine resistance directly limits disease control and survival outcomes for the majority of bladder cancer patients.

Importantly, the clinical significance of gemcitabine does not lie only in how often it is used, but in how often treatment decisions are built around it. A patient may receive gemcitabine intravesically in earlier disease, encounter it again in systemic therapy at a later stage, or be evaluated for combination strategies in which gemcitabine functions as the chemotherapy platform rather than the experimental element. This repeated exposure creates several translational questions. Can resistance be predicted before treatment starts? Can emerging resistance be detected dynamically during therapy? Can drug sensitivity be restored by targeting specific molecular liabilities? These questions are especially relevant now, because the field is moving away from one-size-fits-all chemotherapy and toward a model in which molecular context, disease kinetics, and functional testing are expected to inform therapeutic choice.

Another reason gemcitabine remains difficult to replace is that it occupies a practical middle ground between efficacy, familiarity, and combinability. Clinicians know how to use it, its toxicity profile is relatively well characterized, and it can be paired with platinum, immunotherapy, or radiosensitizing strategies depending on the disease setting (7–10, 12, 13). By contrast, many newer agents are biomarker-selected, cost-sensitive, line-specific, or associated with distinct toxicities that limit universal adoption (11–17). For that reason, the goal is not simply to move beyond gemcitabine. In many patients, the more realistic objective is to identify who is likely to benefit, who is likely to fail early, and where combination or alternative treatment should be considered sooner rather than later.

## 3 Mechanism of action of gemcitabine and the biological starting point of resistance

Gemcitabine is a deoxycytidine analogue whose antitumor activity depends on a sequence of tightly linked intracellular events (18–22). After administration, the drug must first enter tumor cells through nucleoside transporters, particularly equilibrative and concentrative transport systems, of which hENT1 has received the greatest translational attention in several solid tumors (23–27). Once inside the cell, gemcitabine is phosphorylated by deoxycytidine kinase (dCK) to its monophosphate form and then converted into the active diphosphate and triphosphate metabolites (18–21). The diphosphate metabolite inhibits ribonucleotide reductase, reducing the intracellular deoxynucleotide pool, whereas the triphosphate metabolite is incorporated into DNA and leads to masked chain termination, replication arrest, and eventually cell death (18–20). At the same time, gemcitabine can be inactivated by cytidine deaminase (CDA), and changes in transport, activation, or inactivation may each reduce effective intracellular drug exposure (21, 22).

This pharmacologic sequence provides the biological starting point of resistance. In a narrow sense, resistance may begin with reduced transporter expression, diminished dCK activity, or increased CDA-mediated drug inactivation. In a broader sense, however, resistance reflects a layered adaptive process. Tumor cells exposed to gemcitabine do not merely avoid drug entry; they remodel how they repair DNA, manage replication stress, preserve survival signaling, alter metabolic flux, and communicate with surrounding stromal or immune cells (19–22, 28–31). Accordingly, gemcitabine resistance is better viewed as an evolving systems-level phenotype than as a single pathway defect. A Hierarchical Progression Model of Gemcitabine ResistanceTo operationalize the systems level view of resistance, we have established a 3 stage hierarchical model that defines the temporal sequence, parallel interactions, and clinical context dependency of resistance layers, supported by preclinical and clinical data from bladder cancer studies (32–34).

### 3.1 Early pharmacologic resistance

This stage is driven by alterations in the canonical gemcitabine handling pathway, including reduced drug uptake (hENT1 downregulation), impaired activation (dCK loss of function), or enhanced inactivation (CDA/AKR1C3 upregulation). It is typically intrinsic or emerges in the early phase of treatment, and is the dominant resistance mechanism in NMIBC patients receiving intravesical gemcitabine monotherapy (23–27, 33).

### 3.2 Intermediate repair and survival resistance

This stage emerges under sustained gemcitabine exposure, driven by adaptive mechanisms that enable tumor cells to survive drug-induced genotoxic stress. Key pathways include enhanced DNA damage repair (DDR), replication stress tolerance, and activation of pro-survival autophagy and anti-apoptotic signaling (AKT/mTOR). It occurs in parallel with upper stage in MIBC and advanced disease patients receiving systemic GC chemotherapy, and is the dominant mechanism of acquired resistance during active treatment (35–38).

### 3.3 Late cell-state and microenvironmental resistance

This advanced adaptive stage arises after repeated chemotherapy exposure, driven by permanent cell state remodeling and non cell autonomous TME interactions. Key mechanisms include EMT, stemness acquisition, metabolic reprogramming, ncRNA mediated epigenetic regulation, inflammatory TME remodeling, and extracellular vesicle mediated intercellular resistance transmission. It is associated with disease progression, metastasis, and multi-drug resistance, and can reversely amplify resistance in the previous two stages via paracrine signaling (34, 39–41).

Critically, these stages are not strictly linear, they can emerge in parallel in heterogeneous tumors, with crosstalk between layers driving a more aggressive resistant phenotype. This model explains why single layer biomarkers have consistently failed in clinical validation, as they cannot capture the multi factorial, evolving nature of gemcitabine resistance (23–27, 32).

This layered model also helps explain why pharmacologic rescue strategies alone have shown mixed success. Restoring transporter function or inhibiting CDA may improve intracellular drug exposure, but if a tumor has already shifted into a high-autophagy, high-DDR, metabolically plastic state, then correcting one early step may not be sufficient (21, 22, 28–31). Conversely, targeting only a downstream survival pathway may fail if the tumor never accumulates enough active gemcitabine metabolite to generate meaningful stress in the first place. Seen this way, gemcitabine sensitivity depends on the integrity of the whole response chain, from uptake to cell-state adaptation. This is exactly why composite biomarkers are likely to outperform single markers in future clinical studies.

## 4 Molecular network of gemcitabine resistance

**Figure 1:**
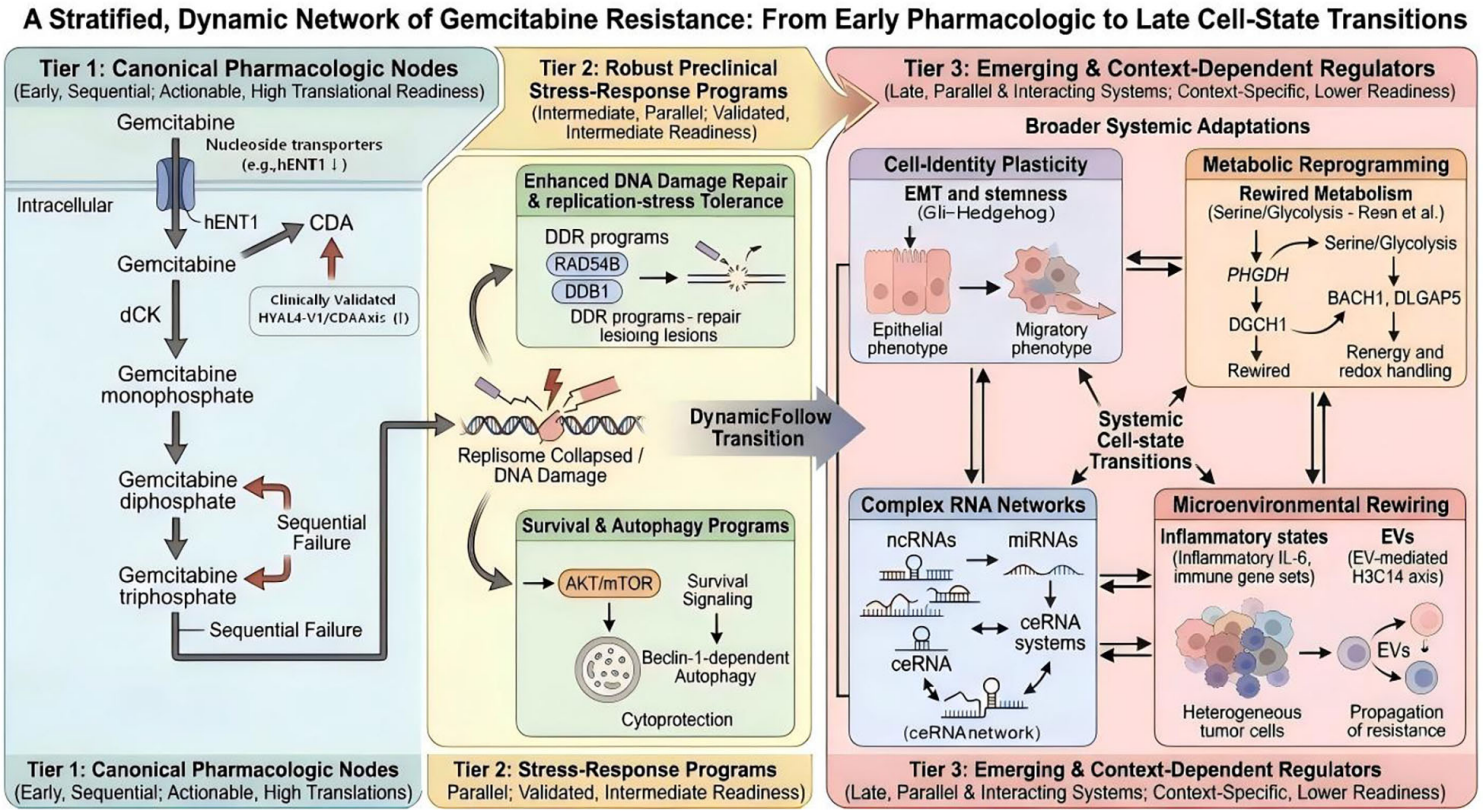
A stratified, dynamic network of gemcitabine resistance.

Current evidence supports a multifactorial, interactive network model of gemcitabine resistance, aligned with the 3 stage hierarchical progression framework defined above. In bladder cancer, resistance arises from the convergence of cell autonomous and non cell autonomous mechanisms, with substantial variation in the quality of clinical evidence and translational readiness across pathways. In the following sections, we summarize these mechanisms from early pharmacologic alterations to late cell state and tissue level regulation, with explicit assessment of clinical evidence priority and translational readiness for each pathway. An integrated hierarchical network of gemcitabine resistance is presented in **Figure 1**, and a summary of key mechanisms, evidence levels, and intervention strategies is provided in **Table 1**.

**Table 1:** Hierarchical stratification and translational roadmap of gemcitabine resistance mechanisms in bladder cancer.

Category (Tier)	Mechanism module	Key molecular nodes	Level of evidence and clinical status	Translational strategy and potential
Tier 1: Canonical Layer (Pharmacologic)	Drug Transport, Activation, and Inactivation	hENT1, dCK, CDA (HYAL4-V1), AKR1C3	High (Clinically Validated): Confirmed in multiple patient cohorts; directly correlates with OS/PFS.	Patient Stratification: Companion diagnostics for hENT1/CDA; Prodrugs: Use of NUC-1031 to bypass transporters.
Tier 2: Adaptive Layer (Preclinical)	DNA Repair (DDR) and Survival Signaling	RAD51, RAD54B, PRPF19-DDB1, AKT/mTOR, Beclin-1 Autophagy	Moderate (Robust Preclinical): Well-characterized in cell lines and PDX models; early clinical signals.	Rational Combinations: Gemcitabine + DDR inhibitors (PARPi/ATRi) or mTOR inhibitors.
Tier 3: Systems Layer (Emerging)	Metabolic Rewiring, ncRNAs, and Microenvironment	PHGDH (Serine pathway), miR-99a, IL-6, Extracellular Vesicles (EVs)	Low (Discovery Stage): Identified via high-throughput omics; clinical relevance requires large-scale validation.	Dynamic Monitoring: Development of urine/blood-based EV liquid biopsy; Target Discovery: Intervening at metabolic vulnerabilities.

### 4.1 Imbalance in drug uptake, activation, and inactivation

From a pharmacologic standpoint, nucleoside transporters, dCK, and CDA remain the canonical resistance nodes (18–27). In other tumor types, hENT1 has repeatedly been explored as a determinant of gemcitabine sensitivity, and although bladder cancer-specific clinical validation remains limited, the same logic is biologically relevant here: reduced intracellular delivery lowers the chance that enough active metabolite will accumulate to damage DNA efficiently (23–27). Meanwhile, enhanced deamination by CDA can sharply attenuate gemcitabine activity. This issue has received renewed attention because resistance-associated pathways in bladder cancer appear capable of upregulating drug-inactivating machinery rather than merely downregulating drug uptake. In that sense, pharmacologic resistance is no longer viewed as passive failure of drug handling; it can be an actively programmed tumor phenotype.

A representative example is the HYAL4-V1/Chase pathway described by Hasanali and colleagues. In their model, the HYAL4 splice variant enhanced CDA expression through CD44-JAK2/STAT3 signaling, thereby promoting gemcitabine inactivation and correlating with chemotherapy failure in bladder cancer (33). The fact that tetrahydrouridine partially restored sensitivity in this setting is important, because it points to a therapeutically actionable node. Likewise, Himura et al. showed that inhibition of AKR1C3 could at least partially reverse gemcitabine/cisplatin resistance (42). Although AKR1C3 is not part of the classical gemcitabine activation pathway, the study reinforces a broader point: enzymes involved in cellular detoxification and metabolic buffering may help sustain the resistant phenotype. Older work on gemcitabine metabolism and newer prodrug strategies such as NUC-1031 further support the idea that bypassing transporter dependence or early metabolic inactivation may offer a rational route to overcoming resistance (28–31).

### 4.2 DNA damage repair and DNA replication stress

Once gemcitabine has triggered replication arrest, tumor cells survive more readily if they can buffer replication stress, restart stalled forks, and repair DNA lesions more efficiently. That is why DNA damage repair (DDR) has become one of the most informative mechanistic layers in recent bladder cancer studies. Liu et al. recently reported that AP1M2 enhances RAD54B-associated DNA repair and drives gemcitabine-cisplatin chemoresistance (35). Yu and Ge further showed that PRPF19 affects gemcitabine sensitivity through DDB1-related regulation of DNA damage repair (36). Together, these studies suggest that resistant cells are not simply repairing more damage in a generic sense; they may be remodeling specific DNA-repair modules that help them survive nucleoside analogue-induced stress.

The relationship between DDR and gemcitabine resistance is also dynamic. Genomic profiling of chemotherapy-resistant MIBC has indicated that post-treatment molecular states do not fully mirror the pretreatment landscape (32). This is clinically relevant. A DDR alteration detected in the resistant setting may represent treatment-driven selection rather than an intrinsic baseline feature. Additional evidence also links RAD51-related repair capacity to gemcitabine response. Gao et al. found that berberine enhanced gemcitabine cytotoxicity through suppression of the PI3K/Akt-RAD51 axis (38). Taken together, these observations support a working model in which gemcitabine resistance emerges through repeated cycles of damage, repair, and clonal selection. From a translational point of view, this is exactly why paired sampling before treatment and at progression is more informative than relying on archival tissue alone.

### 4.3 Survival signaling, apoptosis escape, and autophagy activation

If the DDR network helps explain how bladder cancer cells recover from gemcitabine-induced stress, then AKT/mTOR signaling, autophagy, and apoptosis-related programs help explain how they remain viable long enough to do so. Xiong et al. showed that KNSTRN promotes tumorigenesis and gemcitabine resistance through AKT activation (37). In parallel, bladder cancer cell-intrinsic PD-L1 signaling has been reported to activate mTOR and autophagy, thereby weakening the cytotoxic effect of chemotherapy (43). These findings are conceptually important because they move PD-L1 beyond immune escape and place it within a tumor-cell survival circuit. Put differently, chemoresistance may be reinforced by pathways that are not traditionally classified as drug-metabolism pathways at all.

Autophagy is especially relevant in this context. It may function as a stress-adaptation program that allows tumor cells to endure metabolic and genotoxic injury instead of undergoing apoptosis (44, 45). Several bladder cancer studies support this view. HMGB1-mediated autophagy has been shown to attenuate gemcitabine-induced apoptosis through JNK and ERK activation (46), hypoxia can promote gemcitabine resistance through HIF-1α-dependent autophagy (47), and oblongifolin C was reported to reverse resistance by suppressing autophagic flux (48). More recently, Wang et al. proposed that TPI1 enhances gemcitabine resistance by activating Beclin-1-dependent autophagy (39). OGT-mediated O-GlcNAcylation and Gli-dependent Hedgehog activation also fit within this broader survival framework, linking metabolic sensing, stress response, and aggressive tumor behavior (49, 50). Overall, once autophagy and survival signaling are activated together, the cytotoxic threshold required for gemcitabine to eliminate tumor cells appears to rise substantially.

### 4.4 EMT, stemness, and metabolic reprogramming

Resistant bladder cancer cells often acquire a phenotype that is not only drug tolerant but also more migratory, invasive, and clonogenic. This observation has been reproduced in several models and supports a close relationship between drug resistance, EMT, stemness, and metabolic rewiring. The stepwise chemoresistance model developed by Mun et al. is particularly instructive because it suggests that resistance deepens gradually under sustained drug pressure and that EMT-like changes become more pronounced over time (34). Similarly, Chang et al. showed that gemcitabine can induce Gli-dependent Hedgehog signaling, which helps urothelial carcinoma cells tolerate treatment (49). Such findings imply that repeated chemotherapy exposure may push tumor cells toward a more plastic, stem-like state rather than merely selecting for faster proliferators.

Metabolic reprogramming appears to provide a biochemical foundation for this phenotype. Kawahara et al. identified increased PHGDH expression together with adaptive changes involving IDH2 and HIF1α in gemcitabine/cisplatin-resistant bladder cancer models (51). Subsequent work connected the MINCR/miR-876-5p/ZEB1/PHGDH axis to resistance, again linking serine metabolism to EMT-associated transcriptional control (40). The BACH1/PSPH/S100A2 pathway and DLGAP5-driven MYC stabilization further support the view that rewired glycolysis and amino-acid metabolism help sustain chemoresistance (52, 53). Likewise, NXPH4 has been reported to enhance reactive oxygen species handling and glycolytic activation through NDUFA4L2, thereby promoting gemcitabine resistance (54). In parallel, tumor-derived lactate can fuel the STAT3-LCN2 pathway and reinforce malignant behavior and chemoresistance, linking metabolic by-products directly to adaptive signaling (55). These data collectively suggest that resistance is not only a matter of escaping death; it often reflects a more comprehensive reprogramming of energy production, redox control, and biosynthetic flux.

Stemness-related biology further complicates this picture. Chemotherapy-resistant bladder cancer populations often show features that overlap with cancer stem cells, including enhanced clonogenicity, survival under stress, and a greater capacity for phenotypic switching. These features are not always measured directly in gemcitabine studies, but they help explain why some tumors relapse quickly even after an initial cytoreductive response. Once a minor subpopulation is able to survive treatment and repopulate the tumor, resistance becomes less about one signaling node and more about state transition. LncRNA-mediated repression of differentiation programs, SOX2-related self-renewal control, and EMT-associated transcription factors such as ZEB1 all fit within this framework (40, 56). Thus, the resistant phenotype should not be seen only as a biochemical phenotype. It is also a cell-identity phenotype, and that has implications for treatment durability.

### 4.5 Non-coding RNA and post-transcriptional remodeling

Non-coding RNAs (ncRNAs) provide a critical regulatory layer that coordinates the multi stage resistance network via post-transcriptional and epigenetic fine tuning. In bladder cancer, 3 core functional ncRNA modules have been consistently linked to gemcitabine resistance (56–63):

miRNA Mediated Post Transcriptional Silencing: miRNAs directly target and repress key resistance genes; for example, miR-99a-5p induces cellular senescence in gemcitabine resistant bladder cancer cells by targeting SMARCD1, partially restoring drug sensitivity (57).
lncRNA Mediated ceRNA Networks: lncRNAs act as miRNA sponges to de-repress resistance pathways; for example, GHET1, UCA1, and MINCR drive resistance via sponging of tumor suppressive miRNAs, upregulating ABCC1, ZEB1, and other resistance associated genes (40, 58, 64).
Stemness Associated lncRNA Epigenetic Regulation: lncRNAs mediate epigenetic silencing of differentiation programs to maintain cancer stem cell (CSC) properties; for example, LBCS inhibits self-renewal and chemoresistance of bladder CSCs via epigenetic silencing of SOX2 (56).

At the same time, the field needs to interpret these data carefully. Many ncRNA studies are still based on a limited number of cell lines, short validation chains, or database-driven network construction. Pan et al. nevertheless provided a useful broader view by showing that gemcitabine-resistant bladder carcinoma involves a lncRNA/circRNA coregulated ceRNA system rather than a single dominant RNA axis (59). Other reports suggest that stemness-associated lncRNAs may also influence chemoresistance indirectly by regulating differentiation state and self-renewal capacity, as illustrated by LBCS-mediated epigenetic silencing of SOX2 in bladder cancer stem cells (56). In practical terms, ncRNAs may currently be most valuable as mechanistic clues and candidate biomarker pools. Their transition into robust clinical predictors will require larger cohorts, harmonized platforms, and more consistent demonstration that expression levels remain informative across different treatment settings.

### 4.6 Inflammation, the immune microenvironment, and extracellular vesicles

An increasing number of studies indicate that gemcitabine resistance is not governed solely by tumor cell-intrinsic mechanisms. The inflammatory and immune microenvironment appears to modulate both the emergence and maintenance of the resistant phenotype. Song et al. identified gemcitabine-resistance-associated genes linked to the tumor-immune microenvironment (65), and a retrospective clinical study found that pretreatment serum IL-6 levels were associated with the response to intravesical gemcitabine in T1 NMIBC (66). These findings do not yet establish a complete causal model, but they do suggest that resistant disease may be accompanied by an inflammatory state that is measurable outside the tumor tissue itself. This idea becomes more plausible when one considers that chemotherapy exposure can reshape immune-cell infiltration, cytokine signaling, and stromal interactions over time.

Extracellular vesicles (EVs) provide one mechanism through which this broader microenvironmental rewiring may occur (41, 67–70). EVs can transfer proteins, lipids, RNA species, and chromatin-associated signals between cells, thereby transmitting resistance-associated information rather than confining it to a single clone. Huang et al. recently showed that EV-mediated regulation of H3C14 contributes to gemcitabine resistance in bladder cancer (41). More generally, the expanding EV literature in bladder cancer suggests that vesicle cargo may have dual translational value: it may participate in resistance biology and may also serve as a noninvasive biomarker source in urine or blood (67–70). A summary of representative mechanisms, evidence types, and potential intervention strategies is provided in **Table 1**. Taken together, the microenvironmental perspective broadens the concept of resistance from a cell-autonomous problem to a tissue-level ecosystem problem.

## 5 Potential strategies and future directions for gemcitabine resistance research

Based on the currently available evidence, research on gemcitabine resistance in bladder cancer is moving in four interrelated directions. The first is mechanism-directed reversal of resistance, such as inhibition of CDA, AKR1C3, PHGDH, PSPH, or related metabolic nodes (33, 40, 42, 51, 52). The second is interference with DNA-repair capacity and replication-stress tolerance through pathways involving RAD54B, DDB1, RAD51, or parallel DDR programs (35, 36, 38). The third is suppression of survival circuits, particularly AKT/mTOR signaling, autophagy, O-GlcNAcylation, and Hedgehog activation (37, 39, 43, 46–50). The fourth is a more integrative strategy that combines molecular targeting with biomarker development, microenvironmental modulation, and functional drug testing. This last direction is probably the most clinically meaningful, because even a strong mechanistic target has limited value if the appropriate patient population cannot be identified prospectively.

One practical lesson from the broader gemcitabine literature is that resistance often becomes clinically useful only when it can be translated into a measurable biomarker (18–31). In other cancers, hENT1, dCK, and CDA have all been explored as predictors of response, although reproducibility has varied across platforms and treatment contexts (23–27). For bladder cancer, the analogous challenge is to determine which resistance signals are stable enough to inform treatment decisions and which merely reflect a transient state under drug pressure. The HYAL4-V1/CDA axis is interesting because it combines mechanistic plausibility with a signal in patient material (33). Serum IL-6 is attractive because it is easy to measure (66). Immune-gene signatures, EV-associated molecules, and resistance-linked metabolic markers are also promising (41, 54, 65). Still, none of these markers is ready for routine clinical deployment. To reach that point, future studies need predefined assays, independent validation cohorts, and clinically meaningful endpoints such as pathologic response, progression-free survival, or early progression on gemcitabine-based therapy.

### 5.1 Dynamic biomarkers and liquid-biopsy opportunities

A particularly important direction is dynamic monitoring of resistance rather than one-time baseline stratification. Archival tissue is often a poor surrogate for the molecular state of a tumor that has already been exposed to intravesical therapy, systemic chemotherapy, or immunotherapy. Liquid biopsy therefore offers a way to capture evolving disease biology in real time (16, 69–75). Circulating tumor DNA, urinary tumor DNA, circulating tumor cells, and EV-derived nucleic acids may help identify minimal residual disease, emerging resistant clones, or shifts in pathway activation before overt clinical progression becomes apparent (71–75). For bladder cancer, urine-based approaches are particularly appealing because the disease is anatomically accessible and tumor-derived material may be shed directly into the urinary tract (16, 71, 74). That said, dynamic biomarker development should be tied to specific clinical questions. Predicting failure of intravesical gemcitabine is not the same as predicting resistance in metastatic disease, and the relevant sampling matrix, timing, and cutoff values may differ substantially.

Concrete clinical data already support the translational potential of liquid biopsy in this setting. Urinary tumor DNA (utDNA) detection of CDA promoter hypermethylation has shown 82% sensitivity and 76% specificity for predicting intravesical gemcitabine failure in NMIBC patients, and is now being evaluated in prospective multicenter trials (16, 71, 74). In MIBC patients receiving neoadjuvant GC chemotherapy, post cycle 2 circulating tumor DNA (ctDNA) clearance is an independent predictor of pathological complete response (pCR) and recurrence free survival, with ongoing clinical trials using ctDNA dynamics to guide treatment intensification or de-escalation (72, 73, 75). These examples demonstrate that liquid biopsy is no longer purely aspirational: it is already on the cusp of clinical implementation for guiding gemcitabine treatment in bladder cancer.

### 5.2 Functional precision models and multi-omics integration

Another rapidly developing area is the use of functional patient-derived models. Organoids are especially attractive because they preserve at least part of the histologic and genomic heterogeneity of the parental tumor and can be exposed directly to therapeutic agents ex vivo (15, 76–81). Early bladder cancer organoid studies have shown feasibility for pharmacotyping and for linking drug response to molecular features (76, 77, 79–81). Microfluidic systems add another level of control by recreating three-dimensional architecture, fluid dynamics, and imaging-based response assessment (82). These platforms are not perfect representations of the *in vivo* tumor, but they may capture clinically relevant resistance patterns better than long-passaged cell lines alone. Importantly, their real value will probably emerge when they are combined with genomic, transcriptomic, and phenotypic readouts rather than being treated as stand-alone screening tools.

Concrete clinical validation of organoid pharmacotyping has already been demonstrated in bladder cancer. Multi-center cohorts have shown that patient derived organoids (PDOs) have 83% concordance with patient clinical response to gemcitabine based chemotherapy, and PDOs are now being used in prospective clinical trials to guide second line treatment selection for chemotherapy resistant patients (76, 77, 79–81). Microfluidic 3D models have further improved predictive accuracy by recreating the bladder TME and fluid dynamics, with convolutional neural network based image analysis enabling high throughput drug sensitivity testing (82). These functional models are no longer just research tools: they are emerging as clinically actionable platforms for personalized treatment selection.

The same logic applies to single-cell and spatial technologies. Bulk tissue analysis can identify candidate genes, but it cannot easily resolve whether a resistance signal comes from epithelial tumor cells, stromal fibroblasts, immune infiltrates, or a rare subclone with high plasticity. Single-cell sequencing and spatial transcriptomics are beginning to uncover this level of heterogeneity in bladder cancer (83, 84). Digital spatial profiling, fibroblast-focused spatial studies, and integrated atlases have already shown that the microenvironment of MIBC is highly regionalized and biologically uneven (83, 84). In the context of gemcitabine resistance, such tools may help distinguish between cell-intrinsic metabolic resistance and microenvironment-driven protective niches. That distinction matters, because the therapeutic implications are different: one might require pathway inhibition within tumor cells, whereas the other might call for stromal or immune-directed combination strategies.

There is also a practical advantage to multi-omics integration. When genomic, transcriptomic, spatial, and functional data are interpreted together, they can help prioritize which mechanisms are likely to be causal rather than merely correlative. For example, a metabolic gene that is overexpressed in bulk RNA sequencing becomes more compelling if it is also spatially enriched in aggressive regions, associated with EV-mediated communication, and linked to reduced drug sensitivity in matched organoids. Likewise, an inflammatory marker becomes more useful if it tracks with serum measurements, tissue localization, and clinical outcome. This kind of triangulation will probably be essential in a disease as heterogeneous as bladder cancer, where different resistance routes may coexist within the same patient. In that sense, future biomarker development should be less about finding one perfect molecule and more about constructing an evidence-backed resistance map.

### 5.3 Combination therapy and translational barriers

Combination strategies remain a logical way to delay or reverse resistance. The preclinical literature supports several broad approaches: combining gemcitabine with metabolic inhibitors, autophagy blockers, PI3K/Akt/mTOR pathway inhibitors, inflammatory modulators, or natural compounds that sensitize cells to DNA damage (38, 39, 52, 53, 85–91). Rapamycin-based combination therapy provides at least a preliminary clinical example that resistance-relevant signaling can be targeted in conjunction with cisplatin/gemcitabine (85). Natural products such as berberine, sulforaphane, and ursolic acid are also of interest, not because they are ready-made solutions, but because they illustrate how redox control, survival signaling, and migration-associated phenotypes may be druggable (38, 86, 87). At the same time, rational combination therapy requires a better understanding of sequence, timing, and toxicity. Other preclinical efforts point in the same direction: OIP5-targeted manipulation appears to resensitize bladder cancer cells through the TRIP12-PPP1CB-YBX1 axis (92), pan-RAS inhibition has shown activity in gemcitabine/cisplatin-resistant models (93), and earlier work implicated p38 MAPK signaling in acquired gemcitabine resistance (94). A pathway that appears central in a resistant cell line may not be the best target in patients if the inhibitor cannot be delivered safely or if the relevant biology is present only in a subset of tumors.

Several recurring limitations continue to slow clinical translation. First, many mechanistic studies rely on mixed cisplatin/gemcitabine resistance models, making it difficult to separate gemcitabine-specific effects from broader platinum-associated selection. Second, most datasets remain relatively small and are often generated at a single center. Third, endpoints are inconsistent across studies; some focus on proliferation, others on apoptosis, invasion, stemness, or xenograft growth. Fourth, serially matched pretreatment, on-treatment, and post-progression samples are still rare. As a result, the field often knows which pathways are associated with resistance, but not when they appear, how stable they are, or whether they can truly predict outcome in a clinically actionable way. That gap between mechanistic richness and clinical utility is now the central problem to solve.

By summarizing successful and unsuccessful treatment cases, we propose a 3 tiered roadmap for clinical development:

First-Line Combination: Target Early Pharmacologic Resistance. CDA inhibitor + gemcitabine, for NMIBC patients receiving intravesical therapy, with CDA expression as a predictive biomarker.
Second-Line Combination: Target Intermediate Repair and Survival Resistance. DDR inhibitor (RAD51 inhibitor) or autophagy inhibitor + gemcitabine, for MIBC patients with HRD or autophagy activation, to enhance DNA damage and cytotoxicity.
Third-Line Combination: Target Late Cell-State and Microenvironmental Resistance. Metabolic inhibitor (PHGDH inhibitor) + immune checkpoint inhibitor + gemcitabine, for advanced/metastatic patients with EMT and metabolic reprogramming, to reverse the resistant cell state and reprogram the TME.

### 5.4 Design principles for clinically informative resistance studies

A recurring weakness in the current literature is that mechanistic elegance often exceeds clinical relevance. A clinically informative resistance study should begin with a clearly defined use case: predicting failure of intravesical gemcitabine, identifying poor candidates for neoadjuvant GC, detecting early emergence of resistance in metastatic disease, or selecting salvage combinations after progression. These are related but not interchangeable questions. Each requires different specimens, different timing, and different endpoints. For example, a tissue biomarker linked to pathologic response in MIBC may not be suitable for monitoring intravesical treatment in NMIBC, where urine and serum markers could be more practical. Likewise, a marker measured at progression is not automatically predictive of benefit at baseline. Without this level of design discipline, even biologically interesting markers tend to remain trapped in exploratory studies.

Prospective translational trials should therefore incorporate several elements from the outset. First, sample collection should be longitudinal, not cross-sectional, and should include tumor tissue whenever feasible together with blood, urine, and imaging data. Second, biomarker assays should be analytically standardized early, rather than being redefined after discovery. Third, mechanistic hypotheses should be tested in a functionally relevant model, such as organoids or microfluidic platforms, so that correlation can be separated from causation (76–82). Fourth, study populations should be clinically annotated in enough detail to distinguish resistance arising after intravesical therapy, neoadjuvant chemotherapy, or treatment of metastatic disease. Only under these conditions will it become realistic to decide whether a pathway such as CDA-STAT3, PHGDH-driven serine metabolism, EV signaling, or autophagy activation is merely associated with resistance or can actually guide treatment selection.

In practical terms, future studies should be designed around translational clarity. A useful resistance project should ideally specify the treatment setting, define whether the biomarker is predictive or prognostic, collect paired tissue and liquid samples along the same timeline, and incorporate at least one functional platform to test causality. Integration will be more important than volume. More candidate genes alone will not solve the problem. What the field needs are reproducible mechanistic nodes that can be measured with assays feasible in real-world clinical practice. Blood-based proteins, urinary EV cargo, ctDNA dynamics, spatially defined stromal signatures, and organoid drug-response profiles may eventually converge into a composite model of gemcitabine sensitivity. That kind of model would be far more valuable than a long list of isolated biomarkers that never move beyond discovery cohorts.

## 6 Discussion

The clinical value of gemcitabine in bladder cancer has not disappeared with the arrival of immunotherapy, targeted therapy, or antibody-drug conjugates. On the contrary, gemcitabine remains relevant because it continues to sit at the intersection of intravesical treatment, perioperative chemotherapy, and first-line systemic therapy in advanced disease. What limits its benefit is not a lack of pharmacologic rationale, but the fact that resistance develops frequently and often becomes apparent only after the therapeutic window has narrowed. Over the past few years, research has expanded the concept of gemcitabine resistance from a narrow focus on drug metabolism to a broader, multilayered network involving transporter biology, intracellular inactivation, DNA repair, autophagy, EMT, metabolic rewiring, non-coding RNA regulation, inflammatory signaling, and extracellular-vesicle-mediated communication.

The next step should not simply be the identification of yet another candidate molecule. What matters now is whether these mechanistic findings can be anchored in real patient trajectories and converted into biomarkers that are reproducible, clinically practical, and informative enough to influence treatment decisions. If that transition can be achieved, research on gemcitabine resistance will move beyond descriptive molecular cataloguing and begin to produce tangible benefit for patients with bladder cancer.
